# Structural characterization of the *Plasmodium falciparum* lactate transporter PfFNT alone and in complex with antimalarial compound MMV007839 reveals its inhibition mechanism

**DOI:** 10.1371/journal.pbio.3001386

**Published:** 2021-09-09

**Authors:** Xi Peng, Nan Wang, Angqi Zhu, Hanwen Xu, Jialu Li, Yanxia Zhou, Chen Wang, Qingjie Xiao, Li Guo, Fei Liu, Zhi-jun Jia, Huaichuan Duan, Jianping Hu, Weidan Yuan, Jia Geng, Chuangye Yan, Xin Jiang, Dong Deng

**Affiliations:** 1 Department of Obstetrics, Key Laboratory of Birth Defects and Related Disease of Women and Children of MOE, State Key Laboratory of Biotherapy, West China Second Hospital, Sichuan University, Chengdu, China; 2 State Key Laboratory of Membrane Biology, Beijing Advanced Innovation Center for Structural Biology, Tsinghua-Peking Center for Life Sciences, School of Life Sciences, Tsinghua University, Beijing, China; 3 West China School of Pharmacy, Sichuan University, Chengdu, China; 4 School of Pharmacy, Sichuan Industrial Institute of Antibiotics, Chengdu University, Chengdu, China; 5 Department of Laboratory Medicine, West China Hospital, Sichuan University and Collaborative Innovation Center for Biotherapy, Chengdu, China; 6 School of Biotechnology and Biomolecular Sciences, The University of New South Wales, Sydney, Australia; Deakin University, AUSTRALIA

## Abstract

*Plasmodium falciparum*, the deadliest causal agent of malaria, caused more than half of the 229 million malaria cases worldwide in 2019. The emergence and spreading of frontline drug-resistant *Plasmodium* strains are challenging to overcome in the battle against malaria and raise urgent demands for novel antimalarial agents. The *P*. *falciparum* formate–nitrite transporter (PfFNT) is a potential drug target due to its housekeeping role in lactate efflux during the intraerythrocytic stage. Targeting PfFNT, MMV007839 was identified as a lead compound that kills parasites at submicromolar concentrations. Here, we present 2 cryogenic-electron microscopy (cryo-EM) structures of PfFNT, one with the protein in its apo form and one with it in complex with MMV007839, both at 2.3 Å resolution. Benefiting from the high-resolution structures, our study provides the molecular basis for both the lactate transport of PfFNT and the inhibition mechanism of MMV007839, which facilitates further antimalarial drug design.

## Abstract 2

The malaria parasite’s lactate transporter, PfFNT, is a potential drug target due to its housekeeping role in lactate efflux during the red blood cell stage. Cryo-EM structures of PfFNT alone or in complex with the anti-malarial drug MMV007839 reveal the mechanism of lactate transport and drug inhibition.

## Introduction

Malaria remains a worldwide life-threatening disease, leading to an estimated 229 million infections and 409,000 deaths worldwide in 2019 [1]. *Plasmodium falciparum*, one of the 5 agents of human malaria, is responsible for more than half of the total infections globally and nearly all the deaths in WHO African region, representing the deadliest form of *Plasmodium* spp. Despite advances in chemotherapies in past decades, efforts to eradicate malaria have been hampered by the emergence of multidrug-resistant parasites [2,3], which calls for novel antimalarial agents [4].

**Fig 1:**
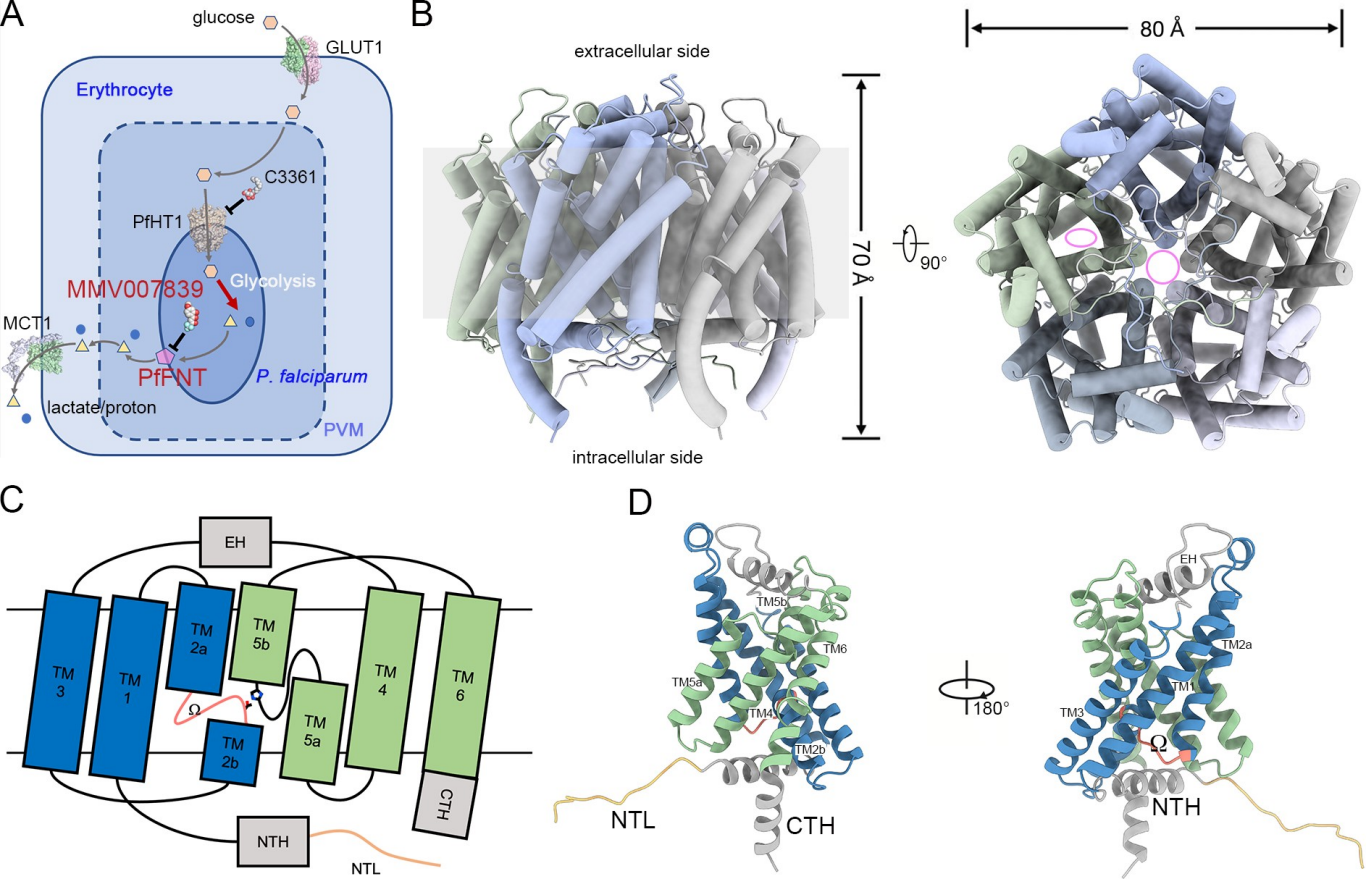
Overall structure of PfFNT. **(A)** Hexose–monocarboxylate transport system of the *P*. *falciparum*–infected erythrocyte. Glucose and lactate are represented by orange hexagons and yellow triangles, respectively. Protons are presented by the blue circles. The magenta pentagon represents the PfFNT protein. Other reported structures, including human glucose transporter GLUT1 (PDB code: 4PYP), human monocarboxylate transporter MCT1 (PDB code: 6LZ0), and *P*. *falciparum* hexose transporter PfHT1 (PDB code: 6M2L), are presented as surface representations. Inhibitors of PfFNT (MMV007839) and PfHT1 (C3361) are displayed as sphere models. **(B)** Overall structure of pentameric PfFNT. The central tunnel of the pentamer and substrate translocation path in the protomer are indicated by pink circle and ellipse in the top view, respectively. **(C)** Topology diagram of a PfFNT protomer. The N-terminal and carboxyl-terminal TM segments are colored blue and green, respectively. Soluble helices, including the EH, NTH, and CTH, are colored gray. The NTL and Ω loops are colored sandy brown and salmon, respectively. Thr106 and His230 are presented as side chain models. **(D)** Cartoon representation of a PfFNT protomer. Components of the protomer are labeled in C. CTH, carboxyl-terminal helix; EH, extracellular helix; GLUT1, glucose transporter 1; MCT1, monocarboxylate transporter 1; NTH, N-terminal helix; NTL, N-terminal loop; PDB, Protein Data Bank; PfFNT, *P*. *falciparum* formate–nitrite transporter; PfHT1, *P*. *falciparum* hexose transporter 1; PVM, parasitophorous vacuole membrane; TM, transmembrane.

The asexual stage of *P*. *falciparum* takes up glucose from host erythrocytes as a primary energy source [5]. In addition, to adapt to the rapid growth and proliferation of asexual stage parasites, *P*. *falciparum* mainly relies on the glycolysis to maintain its energy supply, which results in the fast consumption of glucose and accumulation of lactate [6]. Rapid absorption of glucose and excretion of lactate are executed by the cooperation of 4 essential transporters: human glucose transporter 1 (hGLUT1), *P*. *falciparum* hexose transporter 1 (PfHT1), *P*. *falciparum* formate–nitrite transporter (PfFNT), and human monocarboxylate transporter 1 (hMCT1) (Fig 1A). Briefly, a high concentration of blood glucose enters erythrocytes through facilitated diffusion via hGLUT1 [7], followed by rapid diffusion into *P*. *falciparum* via PfHT1 [8]. After glucose is broken down into lactic acid by glycolysis inside the parasite, the end products, including lactate and protons, are extruded to the extraparasite milieu by PfFNT [9,10]. Finally, hMCT1 mediates the efflux of lactate and protons toward the extracellular space [11].

The *P*. *falciparum* lactate transporter PfFNT, encoded by *pf3D7_0316600*, belongs to the formate–nitrite transporter (FNT) family. As an essential component of the glucose–lactate transport cycle, PfFNT, together with PfHT1, is vital for the energy supply and metabolic homeostasis of parasites [9,10]. The energy supply of parasites can be cut off by inhibiting glucose uptake, making PfHT1 a valuable drug target for next-generation antimalarial development [12–14]. PfFNT, hence, represents another Achilles’ heel of this transport cycle that can be exploited for chemotherapeutic development of malaria. As expected, screening of malaria box, collected by the Medicine for Malaria Venture (MMV), yielded 2 structurally similar PfFNT inhibitors, MMV007839 and MMV000972, which kill parasites at submicromolar and single-digit micromolar levels, respectively [15–17]. Structure–activity relation (SAR) studies and potent characterization of BH296, a derivative of MMV007839, revealed that vinylogous acid rather than cyclic hemiketal is the active form of MMV007839 [15,18].

Despite progress made in inhibitor screening and optimization, structural characterization of FNT family members was limited to prokaryotic homologs. Previously, structures of 5 prokaryotic FNT family members have been reported, including formate channels (FocAs) from *Escherichia coli* [19], *Vibrio cholera* [20], and *Salmonella typhimurium* [21]; nitrite channel (NirC) from *S*. *typhimurium* [22]; and hydrosulphide ion channel (HSC) from *Clostridium difficile* [23]. During our manuscript preparation, structures of nanodisc reconstituted PfFNT in apo form and bound with MMV007839 were reported at 2.6 Å and 2.8 Å, respectively [24]. Due to the relatively low resolution of these 2 structures, the unique N-terminal loop (NTL), which reveals a different transport mechanism than a previously reported prokaryotic model, was not fully characterized. Moreover, the model of MMV007839 was built as a cyclic hemiketal, the prodrug form, rather than the active, vinylogous acid, leading to an incorrect interpretation of the mode of action between PfFNT and the inhibitor. Consequently, we reported 2 cryo-electron microscopy (cryo-EM) structures of PfFNT in apo form or in complex with MMV007839, both at a resolution of 2.3 Å. Our unambiguous ligand density revealed the vinylogous acid model of MMV007839, leading to an accurate and comprehensive understanding of current inhibitor optimization. Our high-resolution structures also revealed the tight coordination between the protomers of the PfFNT pentamer. Combined with the local switching of a bulky residue in the intracellular constriction site, our results indicated an alternating access mechanism different from previously reported N-terminal domain movement in prokaryotic FNT family members.

## Results

### Overall structure of PfFNT

To solve the structure of PfFNT, full-length PfFNT was purified to homogeneity. The cryo-EM images exhibited monodispersed particles, revealing homogeneous protein behavior (S1A Fig). Two-dimensional classification results clearly display the pentameric assembly of PfFNTs that has been observed in structures of bacterial FNT family members (S1B Fig). Using C5 symmetry, we solved the structure of PfFNT to a final resolution of 2.3 Å (Fig 1B, S1 and S2 Figs, S1 Table). Owing to the high resolution, the polypeptide chain, including residues 7 to 293, of each protomer was unambiguously assigned to the density map (S2C Fig). Although 250 mM sodium lactate was incubated with PfFNT before grid preparation, no extra density for lactate was found, which might be attributed to the low affinity between lactate and PfFNT. The 5 protomers of PfFNT resemble as a pentagon-like homopentamer, with a width of approximately 80 Å and height of approximately 70 Å, with a 5-fold axis that is perpendicular to the membrane plane (Fig 1B). Although a hydrophobic central tunnel is encompassed by the 5 protomers, functional characterization of both PfFNT and other FNT family members implies that one tunnel of each protomer, rather than the central tunnel, acts as the pathway for substrate translocation [25,26].

Currently, the structures of 5 prokaryotic FNT family members have been elucidated [19–23,26], and they share approximately 20% identity and 40% similarity with the eukaryotic PfFNTs (S3 Fig). Similar to the reported prokaryotic homologs, each protomer of PfFNT, comprising 6 transmembrane (TM) segments, presents a typical 3+3 invert repeat that is conserved throughout FNT family members (Fig 1C and 1D). The intervening sequence between TM3 and TM4 forms an extracellular helix (EH) that lays on the outer leaflet of the membrane. Two discontinuous helices, TM2 and TM5, enwrap the central pore of each protomer and form constriction sites on both the intracellular and extracellular sides of the membrane. Together with tunnels from the cytoplasmic and extracellular sides, a pathway for substrate translocation is located in the center of each protomer (Fig 1B–1D). To facilitate the comparison with a previously proposed transport model, the loop region between TM2a and TM2b is designated to the Ω loop [20]. Notably, except for the common features of previous prokaryotic homologs, PfFNT processes 2 unprecedented structures, a long and unstructured NTL and an additional carboxyl-terminal helix (CTH) (Fig 1C and 1D). The sequences of the cytosolic NTL, N-terminal helix (NTH), and CTH of PfFNT are not similar to those of prokaryotic homologs but are highly conserved in all *Plasmodium* species, representing the unique features of lactate transporters of parasites (S3 Fig).

### Blocking of the lactate transport path with MMV007839

**Fig 2:**
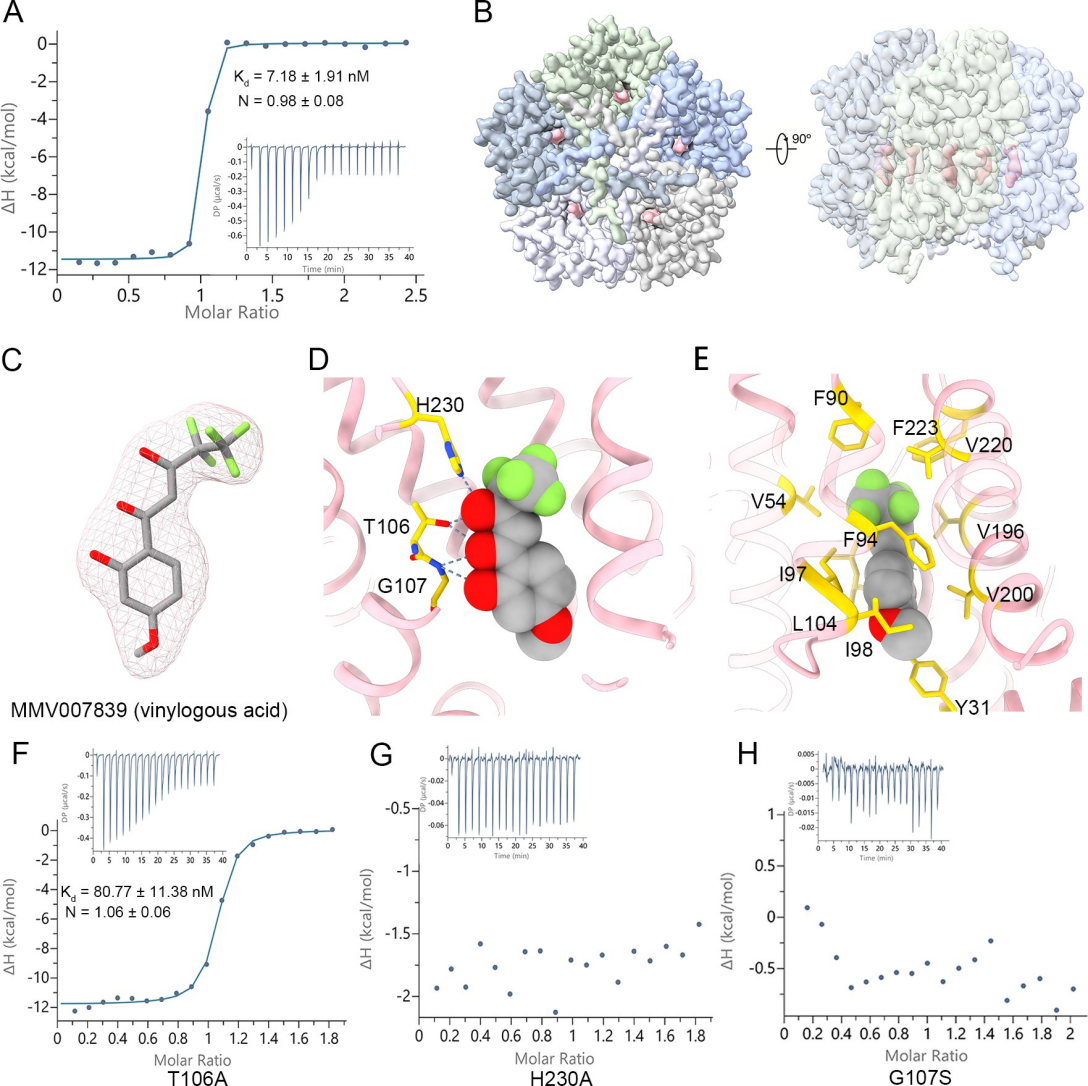
Inhibition of PfFNT by MMV007839. **(A)** Binding affinity between PfFNT and MMV007839. **(B)** Density map of PfFNT in complex with MMV007839. The extra density for MMV007839 is highlighted in pink. The 5 protomers of PfFNT are distinguished by different colors. **(C)** The ligand density fits with the vinylogous acid form of MMV007839. The density for MMV007839, shown as the pink mesh, is contoured at 7.5 σ. **(D)** Coordination between PfFNT and MMV007839. MMV007839 is represented by sphere model. The polar contact between PfFNT and MMV007839 is shown. Inhibitor binding residues are shown as sticks and colored yellow. **(E)** The hydrophobic interactions between PfFNT and MMV007839. The hydrophobic residues in the cavity are shown as sticks and colored yellow. **(F)** Binding affinity between PfFNT _T106A_ and MMV007839. **(G)** Binding affinity between PfFNT _H230A_ and MMV007839. **(H)** Binding affinity between PfFNT _G107S_ and MMV007839. The binding assay was performed via ITC and repeated 3 times. A representative titration is presented. The binding affinity (Kd) and *N* are presented as the value of mean ± SD (S2 Table). The raw data can be found in S1 Data. ITC, isothermal titration calorimetry; PfFNT, *P*. *falciparum* formate–nitrite transporter.

Previous investigations revealed that MMV007839 kills *Plasmodium* parasites at a submicromolar IC50 [15–17]. To validate the in vitro binding affinity between PfFNT and MMV007839, isothermal titration calorimetry (ITC) assays were carried out. The binding affinity between PfFNT and MMV007839 was approximately 7.18 nM (Fig 2A, S2 Table).

To decipher the inhibition mechanism of MMV007839, we incubated 0.047 mM PfFNT with 5 mM inhibitor for 30 minutes to generate PfFNT–MMV007839 complex, followed by cryo-EM sample preparation and data collection. The PfFNT–MMV007839 complex structure was solved at 2.29 Å resolution (Fig 2A, S4 and S5 Figs, S1 Table). The homopentamer structure observed in the PfFNT–MMV007839 complex is similar to that in apo PfFNT. An additional density was observed in the central tunnel of each protomer (Fig 2B). In line with previous biochemical characterization [15,18], the unambiguous inhibitor density perfectly fit with the vinylogous acid form of MMV007839, whereas the prodrug state, the hemiketal form of MMV007839, was smaller and protruded outside the ligand density (Fig 2C, S6A and S6B Fig). Interestingly, recently reported PfFNT–MMV007839 structure built a prodrug form in ligand density due to its limited resolution [24].

Detailed inspection of the inhibitor binding site revealed that MMV007839 inserts its pharmacophore, the fluoroalkyl moiety plus vinylogous acid [15], into the central pore beneath the extracellular constriction site (Fig 2C). The phenol ring of MMV007839 plugs in the intracellular constriction site, making it open toward the cytosolic side. The binding of MMV007839 to PfFNT is mediated by both hydrophilic and hydrophobic interactions. Specifically, 3 polar residues, Thr106, Gly107, and His230, of the central pore form a hydrogen bond network with the vinylogous acid moiety of MMV007839, whereas the rest of the inhibitor contacts the hydrophobic part of the central pore through van der Waals interactions (Fig 2D and 2E). To validate the direct contacts of MMV007839, the single-point mutations (T106A, H230A, or G107S) of PfFNT were generated, and the binding affinities between these mutants and MMV007839 were measured using ITC. As the results showed, the binding affinity of MMV007839 with PfFNT_T106A_ dropped to 80.77 nM, which is an order of magnitude lower than that of wild-type PfFNT (Fig 2F, S2 Table). Notably, the binding affinity of MMV007839 with PfFNT_H230A_ or PfFNT_G107S_ was undetectable (Fig 2G and 2H, S2 Table). Our results indicate that MMV007839 seals the central pore of the substrate transport path, inhibiting the transport activity of PfFNT.

The tight coordination between MMV007839 and surrounding residues of the binding pocket illuminates previous SAR studies that intended to improve the potency of lead compounds based on MMV007839 scaffold [15,18]. Briefly, the modification of the hemiketal moiety, which locks the derivatives into a prodrug form, leads to incompatibility of the ligand with the substrate binding pocket. Shortening of the fluoroalkyl chain decreases the area of the hydrophobic interface enclosed by Val54, Phe90, Val 196, Val220, and Phe223, whereas substantially elongated fluoroalkyl chain to nonafluorobutyl chain cause steric effect due to the closed state of the extracellular constriction site. In contrast, substitution of the methoxy group to decrease or increase the volume of the aromatic tail leads a moderately changes the affinity because the intracellular tunnel is opened and can then accommodate the additional bulk group.

### Mechanistic insight into the drug-resistant mutant G107S

Two independent groups have reported that constantly exposing *P*. *falciparum* to MMV007839 results in mutagenesis of Gly107 to Ser in PfFNT, which leads to an inhibitor-insensitive parasite and a drug-resistant phenotype [15,16]. In the structure of inhibitor-bound PfFNT, an amide nitrogen directly coordinates with the carbonyl group of the vinylogous acid moiety and the hydroxyl group of the phenol moiety (Fig 2D). Additional hydroxymethyl side chain in the G107S replacement mutation substantially increase the steric hindrance of MMV007839, decreasing inhibitor affinity.

To circumvent the drug-resistant mutation, a series of MMV007839 derivatives have been synthesized and characterized through SAR studies [15,18]. Notably, a pyridine substitution of the original phenol moiety yielded the most potent dual inhibitor, BH267.meta, to both wild type and PfFNT_G107S_. In addition to its high efficacy in killing cultured *P*. *falciparum* parasites, BH267.meta prevented the formation of drug resistance even when the parasite was treated with it for a long period [18]. Moreover, a recent study revealed that BH267.meta inhibits a broad spectrum of FNTs, including all causal agents of human malaria, indicating its potential application in further chemotherapeutic development [27].

**Fig 3:**
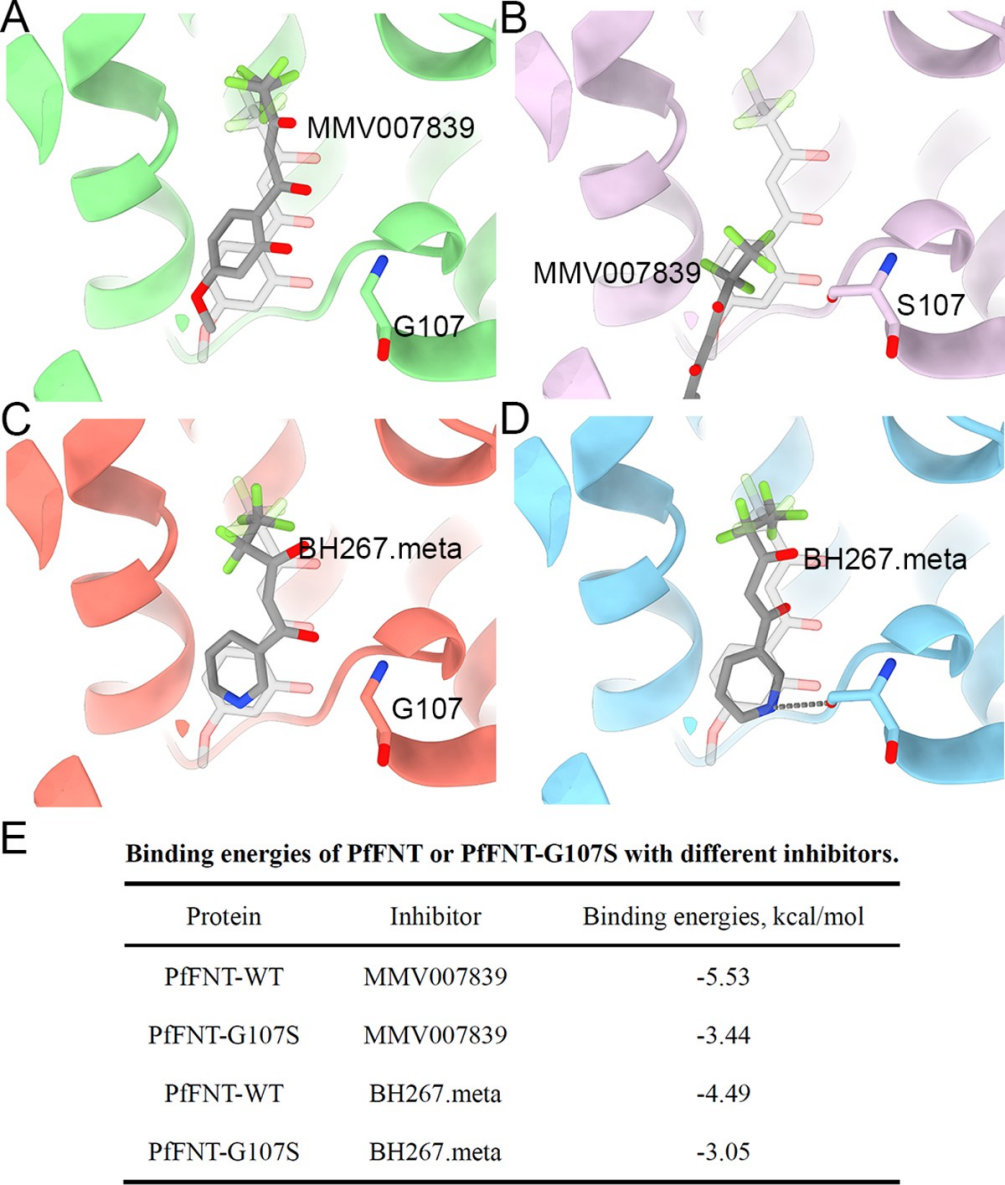
Molecular docking of MMV007839 and BH267.meta in the wild-type PfFNT or G107S resistant mutant. **(A)** The docking model of MMV007839 in the wild-type PfFNT. **(B)** The docking model of MMV007839 in PfFNT_G107S_. **(C)** The docking model of BH267.meta in the wild-type PfFNT. **(D)** The docking model of MMV007839 in PfFNT_G107S_. **(E)** Summary of binding energy of docking results. The transparent stick model of MMV007839 represents the real location in the inhibitor-bound structure. The structure of PfFNT is presented as cartoon representation and Gly107 or S107 are presented as sticks. The docking compounds are represented as sticks and colored gray. PfFNT, *P*. *falciparum* formate–nitrite transporter; WT, wild type.

To elucidate the mechanism by which BH267.meta circumvents the G107S resistance mutation, we docked MMV007839 and BH267.meta to both the wild-type and PfFNT_G107S_ models (Fig 3). Consistent with the complex structure, MMV007839 docked into the inhibitor binding pocket of wild-type PfFNT with a slight shift in its location (Fig 3A). However, introduction of a hydroxymethyl side chain by mutating Gly107 to Ser inhibited MMV007839 by sterically blocking the entrance of the inhibitor to the central pocket (Fig 3B). Consistent with this model, the binding affinity between MMV007839 and PfFNT_G107S_ was undetectable (Fig 2H). BH267.meta, by contrast, could be bound to both the wild-type PfFNT and the G107S mutant (Fig 3C and 3D). The major discrepancy in docking results between MMV007839 and BH267.meta is caused by 2 reasons: (1) the removal of the hydroxyl group of the aromatic moiety avoids the inhibitor clashing with the side chain of Ser107; and [2] a novel hydrogen bond is introduced between the pyridine group of BH267.meta and the hydroxymethyl group of Ser107. Nonetheless, the Gly107➔Ser mutation still increases steric hindrance of BH267.meta, leading to a slight increase in binding energy (Fig 3E). This result is consistent with the report that the affinity of BH267.meta to the G107S mutant is slightly lower than that of wild-type PfFNT [18].

### Structural comparison between apo and inhibitor-bound PfFNT

**Fig 4:**
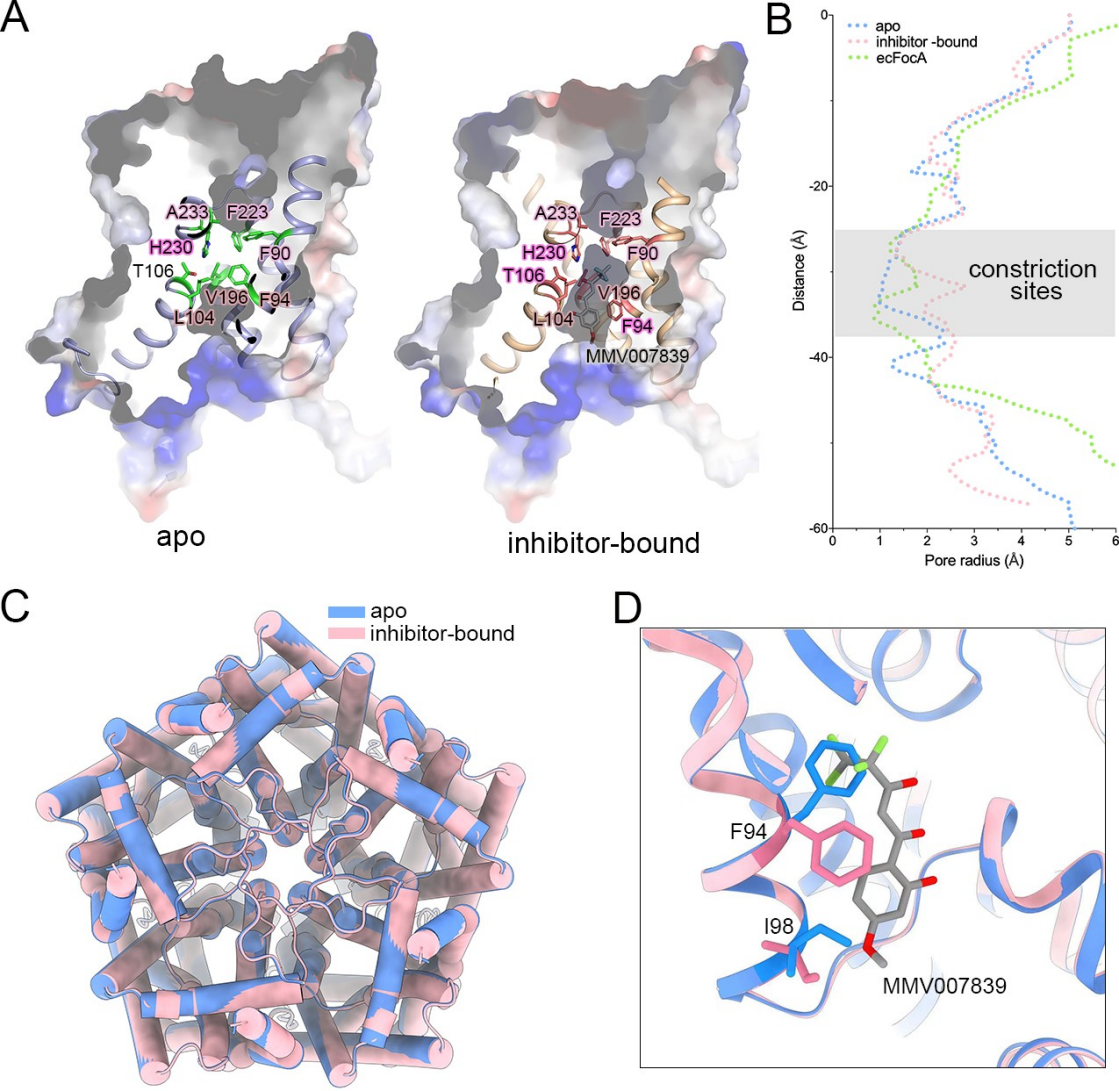
Structural comparison between apo form and inhibitor-bound PfFNT. **(A)** The constrictive site of a PfFNT protomer. The surface potential models of PfFNTs are presented. The residues near the constrictive site are shown as sticks. **(B)** The channel passages of PfFNTs and ecFocA were calculated using HOLE. The region containing the constriction sites is overlapped by transparent gray rectangle. The raw data can be found in S3 Data. **(C)** The superposition of pentameric PfFNT in 2 states. The apo PfFNT and inhibitor-bound PfFNT are colored blue and pink, respectively. **(D)** A comparison of the binding pocket for MMV007839. The inhibitor molecule is shown as grey stick. The Phe94 and Ile98 are represented as sticks. The apo PfFNT and inhibitor-bound PfFNT are colored the same as panel C. ecFocA, *E*. *coli* FocA; PfFNT, *P*. *falciparum* formate–nitrite transporter.

The tunnel for substrate translocation of each PfFNT protomer contains 2 constriction sites. The extracellular constriction site, consisting of Phe90, Phe223, and Ala233, is identical in the sequence of prokaryotic and *Plasmodium* homologous of PfFNT homologs. The intracellular constriction site is formed by 2 conserved residues, Leu104 and Val196, and a variable Phe94 (Fig 4A, S3 Fig). We calculated the radius of the substrate translocation path using HOLE [28]. Similar to *E*. *coli* FocA (ecFocA), the pore radii of the extracellular constriction site for both apo and inhibitor-bound PfFNT are less than 1.5 Å, suggesting closure of the extracellular gate. However, a significant difference was observed around the intracellular constriction site. The apo PfFNT tightly seals at this site with a pore radius of approximately 1 Å, whereas the inhibitor-bound structure opens to the cytoplasmic side (Fig 4A and 4B).

The central pore between 2 constriction sites is encompassed by hydrophobic residues except for 2 polar residues, Thr106 and His230, which are highly conserved in both prokaryotic and *Plasmodium* homologous (S3 Fig). These 2 residues coordinate with each other through a hydrogen bond in the apo form PfFNT (Fig 4A).

We superimposed the structures of the apo and inhibitor-bound PfFNT to examine the gating mechanism. Unlike previously reported cases of prokaryotic FNT family members, substantial conformational change was not observed between the apo state and inhibitor-bound PfFNT. The overall PfFNT structures of the 2 states are primarily identical, with a root–mean–square deviation (RMSD) of 0.19 Å over 287 Cα atoms (Fig 4C). The major discrepancies between these 2 states are the significant side chain shifts of Phe94 and Ile98 (Fig 4D). In particular, phenyl group of Phe94, which constitutes the intracellular constriction site together with Leu104 and Val196, swings away from its original position to avoid collision with the fluoroalkyl moiety of MMV007839, leading to the opening of the intracellular gate (Fig 4A, 4B, and 4D). The movement of the side chain of Ile98, on the other hand, decreased steric hindrance of the methoxy group at the end of MMV007839 (Fig 4D). Taken together, our results suggested that the swing of a bulky residue of the constriction site, exemplified by Phe94, is the key of gating mechanism.

### The unique intracellular region of PfFNT

**Fig 5:**
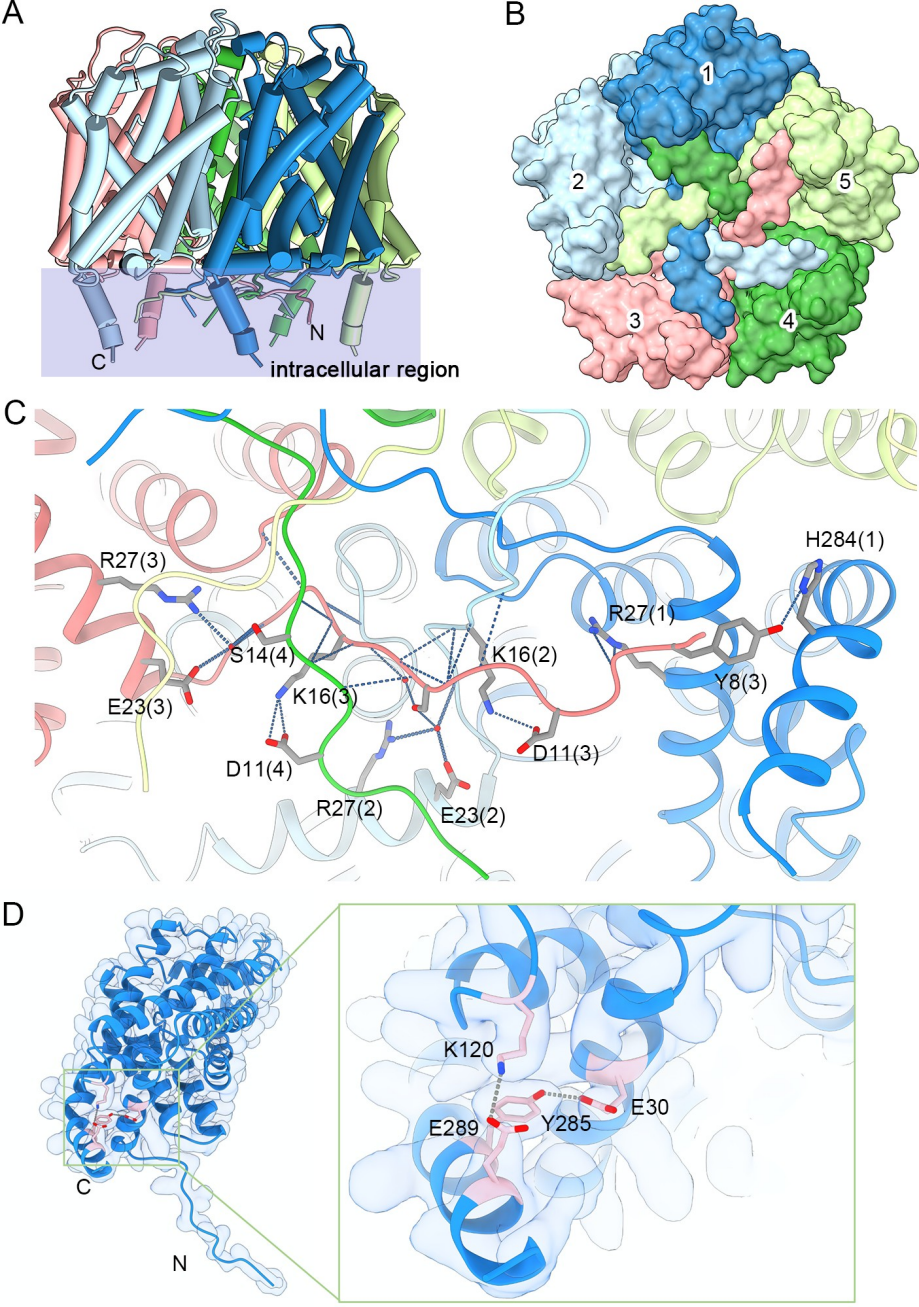
Intracellular region of PfFNT. **(A)** A unique intracellular region is presented in a side view of PfFNT. The overall structure of PfFNT is shown as a cylinder cartoon. The region containing the intracellular region overlaps with a transparent light blue rectangle. **(B)** Overlap of NTLs in a bottom view of PfFNT. The overall structure of PfFNT is shown as a surface, and the 5 protomers are distinguished by different colors. **(C)** The interactions between NTLs. Each protomer is colored the same as in patterns A and B. Hydrogen bonds are shown as the gray dashed lines. **(D)** Coordination between the cytosolic CTH and the rest of the PfFNT protomer. The structure of the protomer of PfFNT is shown as cartoon and fitted to the cryo-EM density. The residues involved in the interactions are shown as pink sticks. Hydrogen bonds are shown as gray dashed lines. cryo-EM, cryo-electron microscopy; CTH, carboxyl-terminal helix; NTL, N-terminal loop; PfFNT, *P*. *falciparum* formate–nitrite transporter.

Consistent with other FNT family members, both the N-terminus and carboxyl terminus of PfFNT are located on in the cytosolic side. Nonetheless, compared with structures of prokaryotic homologs, a unique long NTL and an extra soluble CTH were characterized in our structures (Fig 5A). Although the NTL is unstructured, 5 NTLs of each protomer orderly tangle to each other (Fig 5B). To depict the sophisticated interaction between 5 NTLs, we illustrate the interaction between NTLs from protomers 1, 4, and 5 (Fig 5C). These unstructured NTLs are stabilized by tightly interactions, suggesting a relatively static state of the N-terminal structure. Therefore, the gating mechanism of PfFNT is unlikely to adopt to the previously reported N-terminal reorientation in *S*. *typhimurium* FocA (stFocA) [21], which further supports the aforementioned bulky residue swing model (Fig 5D). The extra CTH protrudes into the cytoplasm of the parasite and is stabilized by 3 hydrogen bonds (Tyr8-His284, Glu30-Tyr285, and Lys120-Glu289; Fig 5C and 5D). Sequence alignment between *Plasmodium* and prokaryotic FNTs reveals that the NTL, NTH, and CTH regions, especially for residues involved in NTL/CTH interactions, are highly conserved in *Plasmodium* spp. but vary in prokaryotic homologous (S3 Fig). In addition, the Lys120 from the loop region between TM2 and TM3 is also highly conserved throughout *Plasmodium* species. Therefore, the intracellular region of PfFNT, including the unstructured NTL and protruding CTH, represents the unique feature of FNTs in *Plasmodium* parasites.

### Structural comparison between PfFNT and prokaryotic FocA

**Fig 6:**
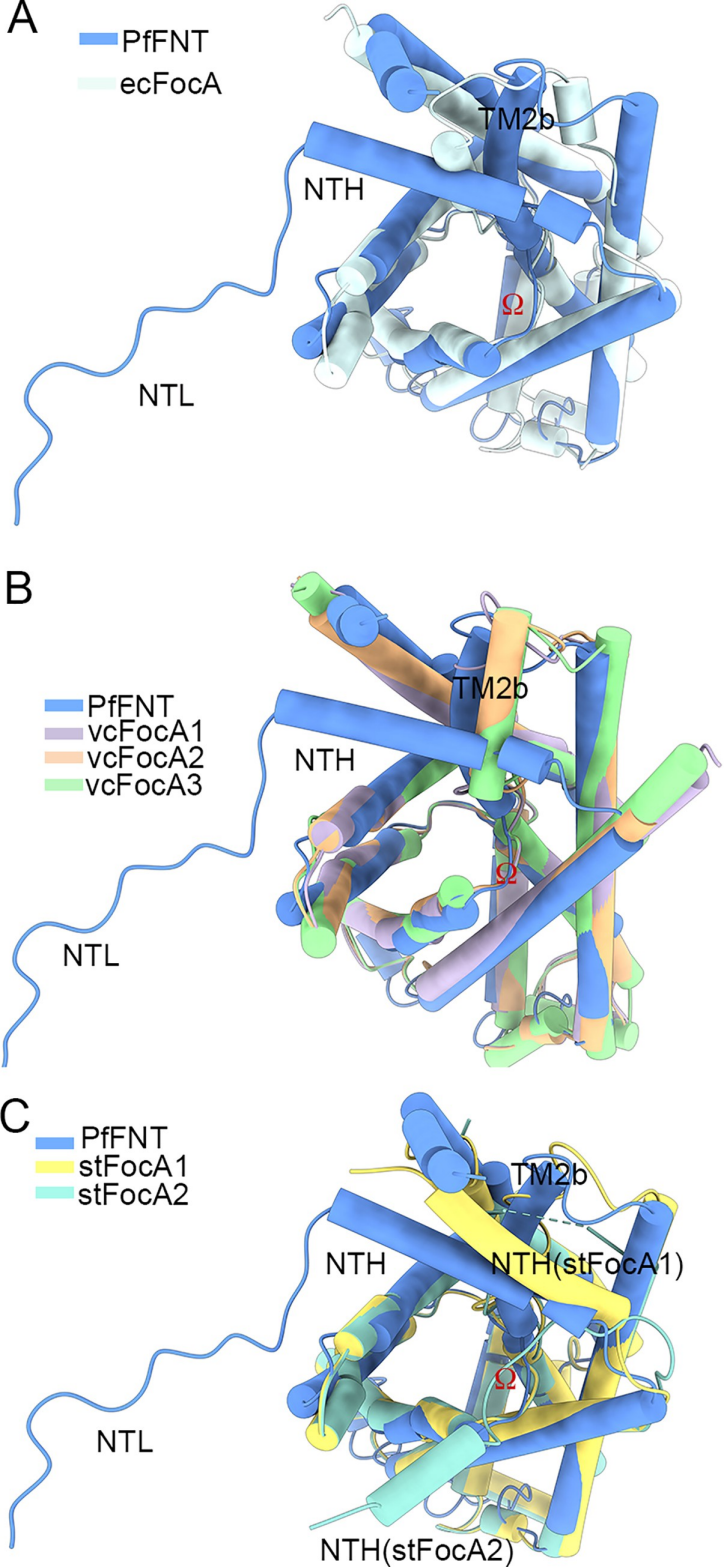
Structural comparison between PfFNT and prokaryotic FocA. **(A)** Comparison of the protomer structures of PfFNT and ecFocA. A discrepancy between PfFNT and ecFocA is observed in TM2b. **(B)** Comparison of the protomer structures of PfFNT and vcFocA. A discrepancy between PfFNT and vcFocA is observed in TM2b. **(C**) Comparison of the protomer structures of PfFNT and stFocA. The NTH of PfFNT performs a different function than that of stFocA. All protomers of FNT proteins presented here exhibited a nearly identical overall structure, with a RMSD of approximately 0.9 Å over 139 to 174 Cα atoms. ecFocA, *E*. *coli* FocA; FNT, formate–nitrite transporter; FocA, formate channel; NTH, N-terminal helix; NTL, N-terminal loop; PfFNT, *P*. *falciparum* formate–nitrite transporter; RMSD, root–mean–square deviation; stFocA, *S*. *typhimurium* FocA; TM, transmembrane; vcFocA, *V*. *cholera* FocA.

Previous structural characterizations of prokaryotic FocA, including ecFocA from *E*. *coli*, *V*. *cholera* FocA (vcFocA) from *V*. *cholera*, and stFocA from *S*. *typhimurium*, have suggested different mechanisms of gating and substrate recognition [19–21]. To further explore the transport mechanism, we carried out extensive structural comparisons between the protomer of PfFNT and prokaryotic FocA (Fig 6). Except for the aforementioned unique cytosolic NTL and CTH, the major discrepancy between PfFNT and ecFocA is observed in TM2b, which also occurs in the structure of vcFocA (Fig 6A and 6B). Interestingly, 2 positions (up and down) of the Ω loop were observed in vcFocA, which was proposed to be essential for opening of its selective filter [20]. However, TM2b of PfFNT is locked by the stable NTH, making it unlikely to transfer between the helix and disorder loop (Fig 6B). Furthermore, the NTH of PfFNT performs a different function than stFocA (Fig 6C). It was proposed that the NTH of stFocA undergoes pH-dependent conformational reorientation during gating [21]. The NTH of PfFNT, however, directly interacts with the CTH and is further stabilized by tangling with the NTL, which locks the NTH into a fixed position (Fig 5C and 5D). Consequently, the unique intracellular region of PfFNT blocks potential movement of the NTH and TM2b. Considering the neutral pH in the cytosol of intraerythrocytic *Plasmodium* parasites [29], the gating mechanism of PfFNT may be quite different from that of the aforementioned bacterial FocA channels. Considering this and the swing of Phe94 between apo and inhibitor-bound PfFNT, we suggest that the shift of the side chains of bulk residues in constriction sites leads to the passage of substrate [30].

## Discussion

The *P*. *falciparum* lactate transporter PfFNT is an essential component of the glucose–lactate transport cycle for intraerythrocytic *Plasmodium* parasites. We and other groups have elucidated the molecular mechanism of the remaining components of this cycle, including the human glucose transporters [31,32], the *Plasmodium* hexose transporters [13,33], and the human monocarboxylate transporters [34,35], leaving the molecular mechanism of PfFNT a mystery.

Recently, Lyu and colleagues reported 2 cryo-EM structures of nanodisc reconstituted PfFNT, revealing a similar protomer structure as FocA and the overall pentamer state [24]. However, due to the limited resolution, both the N-terminal and carboxyl-terminal region are incomplete in their structure. Our high-resolution structures reveal the tight tangling of the NTL, NTH and CTH, indicating a fixed N-terminal region and a different transport mechanism than other FNT members (S7A Fig). Moreover, due to the weak ligand signal, Lyu and colleagues built a prodrug form of MMV007839 in their ligand-bound PfFNT structure, in contrast to previous SAR characterization of MMV007839 derivatives [15]. Benefiting from our high-resolution structure, we elucidated the accurate mode of action between PfFNT and MMV007839 (S7B Fig). We succeeded in identifying essential polar interactions between the inhibitor and central cavity residues and validated those contacts through an in vitro binding assay (Fig 2, S6 Fig), which paves the way to further lead optimization. In addition, we deciphered the resistance mechanism of the PfFNT_G107S_ mutant using our accurate model. Given that the active form of MMV007839 was suggested to mimic 2 consecutive lactate molecules [15], the recognition of the vinylogous acid moiety of MMV007839 by Thr106 and His230 implies a potential substrate recognition mechanism of PfFNT (Fig 2). Our findings are consistent with the observations that corresponding residues (H208 and T90) of vcFocA acted as the formate binding site in previous reports [25,26] and that His230 is essential for substrate transport of FNTs [24,36].

By comparing the structures of PfFNT in 2 different states, we found the conformational changes in the side chains of central cavity residues, especially the rearrangement of Phe94, which forms the intracellular constrictive site. The rearrangement directly leads to the opening of the intracellular gate (Fig 4). Interestingly, 2 conserved phenylalanines (Phe90 and Phe223) form an extracellular constriction site (S3 Fig). Most likely, in a similar way as intracellular gating residue Phe94, side chain movement of these phenylalanines might be related to the gating mechanism of the extracellular gate.

The passage of lactate through PfFNT is coupled to proton transport [10]. Two distinct models of proton coupling have been proposed [24,30,36,37], including the dielectric shift model and the proton transfer model. The dielectric shift model proposed that the substrate is protonated by bulk solvent as the dielectric shift of substrate acidity. The neutralized substrate rather than the anion form is transported by the FNT symporter. On the other hand, proton transfer model referred His230 of PfFNT and its counterpart in other FNT members as a protonation site in the hydrophobic central cavity. The anion is neutralized by a proton donated by His230 for cotransportation. The dielectric shift model of substrate export of PfFNT was deduced on the basis of the positive residues at the cytoplasmic side of PfFNT [37]. Meanwhile, a previous molecular dynamic simulation of FocA suggested that substrate transport is energetically more favorable when the conserved histidine is protonated [38]. We tend to favor the dielectric shift model. However, due to the lack of direct observation of the substrate in our structure, further investigation is needed to reveal the details of the proton coupling mechanism.

Previous studies are controversial regarding whether FNT family members function as transporters or channels, whereas some channels, such as the CLC channel, have been redefined as transporters [39]. We captured an occluded apo form and an inward-open inhibitor-bound state. Through structural comparison between these 2 states and other FocA structures, we elucidated that the substrate is recognized by Thr106 and His230 in the central pore. In addition, combined with the tightly tangled NTL and the swing of Phe94 in the intracellular constriction site, we proposed a bulk residue swing model for gating of PfFNT. In our opinion, the discrepancy between a channel and a transporter is that a channel can simultaneously open both side of gates to allows the continuous flow of substrate, whereas transporter only alternately open to each side of the membrane to allow the access of substrate [40]. Current structures cannot clearly define the whole transport cycle of FNT family members. Therefore, molecular dynamic stimulation may improve our understanding of monocarboxylate transport by PfFNT. Additionally, capturing of other conformational states of PfFNT will facilitate the deciphering of elusive transport mechanisms.

## Methods

### Protein expression and purification

The codon-optimized cDNA of the lactate transporter of *P*. *falciparum*, PfFNT (UniProt No. O77389), was synthesized (Sangon Biotech, China) and subcloned into the PfastBac1 vector with the N-terminal Strep-tag (WSHPQFEK). The Bac-to-Bac expression system (Invitrogen, USA) was used to express the recombinant PfFNT proteins in Sf-9 insect cells [41]. Briefly, the bacmid was generated in *E*. *coli* stain DH10Bac, and the Sf-9 insect cells were used for baculovirus generation and amplification. For protein purification, the Sf-9 insect cells (1.5 × 10^6^ cells/ml) were collected after 72 hours of viral infection and then homogenized in the buffer containing 100 mM Tris pH 8.0, 150 mM NaCl and 1 mM EDTA. For membrane protein extraction, 2% (w/v) n-dodecyl-b-D-maltoside (DDM, Anatrace, USA) and protease inhibitors (PMSF at 1 mM, aprotinin at 0.8 μM, pepstatin at 2 μM, and leupeptin at 5 μg/ml) were added and incubated at 4°C for 3 hours. The soluble fraction was collected by centrifugation (100,000 g, 30 minutes at 4°C) and incubated with Strep-Tactin XT resin (IBA Lifesciences, Germany) at 4°C for 30 minutes. The resin was rinsed with the buffer containing 100 mM Tris pH 8.0, 150 mM NaCl, 1 mM EDTA and 0.06% (w/v) glyco-diosgenin (GDN, Anatrace). The PfFNT proteins were eluted with the buffer containing 100 mM Tris pH 8.0, 150 mM NaCl, 1 mM EDTA, 100 mM biotin (IBA Lifesciences) and 0.06% GDN. The eluted proteins were concentrated to 10 mg/ml and applied to Superose 6 increase (GE Healthcare, USA) with the buffer containing 25 mM Tris pH 8.0, 150 mM NaCl and 0.06% GDN. The PfFNT proteins were further concentrated to 7 mg/ml for cryo-EM sample preparation.

### Isothermal titration calorimetry

The binding assay was performed using a PEAQ ITC (Malvern Panalytical, UK) at 25°C. The concentration of PfFNT protein was adjusted to approximately 10 to 20 μM (protomer). The wild-type PfFNT and its mutants were purified as described above. The peak fractions from the gel filtration were collected and diluted to approximately 10 to 20 μM as a monomer with the gel filtration buffer (with 0.2% DMSO). The inhibitor MMV007839 was dissolved in DMSO at a concentration of 100 mM and then diluted to a final concentration of 200 μM with gel filtration buffer (with 0.2% DMSO). The inhibitor was injected 19 times (0.4 μl for injection 1 and 2 μl for injections 2 to 19), with 120-second intervals between injections. The titration data were analyzed using a one-site binding model, and the first injection was removed. The titration of MMV007839 into the buffer was deducted. All the experiments were repeated 3 times. A representative result is shown, and the binding affinity (Kd) and *N* are presented as the mean ± SD.

### Cryo-EM sample preparation and data acquisition

The purified PfFNT protein was incubated with 250 mM sodium lactate (Sigma-Aldrich, USA) or 5 mM MMV007839 (IBS, Montenegro) for 30 minutes and centrifuged before being applied to the grids. Three microliters of the protein sample was placed on glow-discharged holey carbon grids (Quantifoil Au R1.2/1.3, 300 mesh). The grids were blotted for 3.0 seconds and flash-frozen in liquid ethane cooled by liquid nitrogen with a Vitrobot (Mark IV, Thermo Fisher Scientific, USA). The plunger was operated at 8°C with 100% humidity. Grids were transferred to a Titan Krios electron microscope operating at 300 kV and equipped with a Gatan K3 Summit detector and a GIF Quantum Energy Filter (slit width 20 eV) for data collection. Micrographs of PfFNT with substrate or inhibitor were both recorded in the superresolution mode with a calibrated pixel size of 0.3373 Å. Each stack of 32 frames was exposed for 8 seconds. The total dose rate was approximately 5.2 e^−^/sec/Å^2^ for each stack. AutoEMation was used for the fully automated data collection [42]. All 32 frames in each stack were aligned and summed using the whole-image motion correction program MotionCor2 [43] and binned to a pixel size of 0.6746 Å. The defocus value of each image, which was set from −1.5 to −2.0 μm during data collection, was determined by Gctf [44].

### Data processing

For the PfFNT–apo dataset, 875,415 particles were automatically picked by Gautomatch (developed by Kai Zhang, https://www2.mrc-lmb.cam.ac.uk/research/locally-developed-software/zhang-software/#gauto) from 5,568 micrographs. Two rounds of 2D classification were performed using cryoSPARC [45], and a small subset of the selected particles from 2D classification was used to generate the initial model. After 3D heterogeneous refinement and nonuniform refinement, particles from the best classes were merged, and duplicated particles were removed, resulting in a dataset of 253,458 particles with the pixel size of 1.34 Å, which yielded a resolution of 2.88 Å. Particles were then recentered and reextracted using a pixel size of 0.6746 Å by RELION [46]. The particle stack was brought back into CryoSPARC for 1 round of heterogeneous refinement. The best set of particles and model was then subjected to several rounds of nonuniform refinement, local refinement, local contrast transfer function (CTF) refinement and global CTF refinement, resulting in a 2.29 Å map (S1 Fig).

For the PfFNT–MMV007839 dataset, 2,093,320 raw particles were extracted from 5,519 micrographs using cryoSPARC [45]. Several rounds of guided multireference classification were performed using cryoSPARC, during which the good reference used in all the classifications was updated with a better reference whenever possible. After nonuniform refinement, a total of 275,452 good particles were selected, recentered, and reextracted using a pixel size of 0.6746 Å by cryoSPARC. After an additional round of guided multireference 3D classification and several rounds of nonuniform refinement and local CTF refinement, 291,994 particles gave rise to a reconstruction at an average resolution of 2.29 Å (S4 Fig).

The resolution was estimated with the gold-standard Fourier shell correlation (FSC) 0.143 criterion [47]. The angular distributions of the particles used for the final reconstruction of the PfFNT complex are reasonable (S2 and S5 Figs).

### Model building and refinement

The atomic coordinates of the PfFNT were generated by an artificial intelligence (AI)-guided deep natural network method named CryoNet (https://cryonet.ai). The structure was then docked into the density map and manually adjusted and rebuilt by COOT [48]. Three-dimensional structures and restraint files for the inhibitor MMV007839 were generated by the sketcher program in the CCP4 suite [49].

The initial model refinements of PfFNT–apo and PfFNT–MMV007839 were carried out by PHENIX [50] against the corresponding map in real space with secondary structure and geometry restraints. Overfitting of the model was monitored by refining the model in one of the 2 independent maps from the gold standard refinement approach and testing the refined model against the other map [51]. The structures of the PfFNT were validated through examination of the MolProbity scores and statistics of the Ramachandran plots (S1 Table). Molprobity scores were calculated as described [52].

### Molecular docking

Docking studies were attempted to explore the binding mode of the MMV007839 and BH267meta onto the 3D structure of PfFNT using AutoDock tools 4.2.6 [53]. Before docking, polar-H atoms were added to the PfFNT structure, followed by Gasteiger charge calculation using AutoDock tools. The macromolecule file was then saved in pdbqt format and ready to be used for docking. Ligand centered maps were generated by the AutoGrid program with grid dimensions of 130 ×140 × 120 Å. PyMOL package was used to visualize the binding interactions between these ligands and the 3D structure of PfFNT.

## Supporting information

S1 Fig Cryo-EM structural determination of PfFNT. **(A)** A representative cryo-EM micrograph of PfFNT. **(B)** A representative 2D classification average. **(C)** Flowchart for EM data processing of PfFNT datasets. Details can be found in the Methods. cryo-EM, cryogenic-electron microscopy; EM, electron microscopy; PfFNT, *P*. *falciparum* formate–nitrite transporter.
(TIF)

S2 Fig Cryo-EM analysis of the PfFNT structure. **(A)** Gold standard FSC curve for the 3D refinement of the overall structure of PfFNT. The raw data can be found in S3 Data. **(B)** Angular distribution of the particles used for the final reconstructions. **(C)** Local resolution of the PfFNT complex estimated by Chimera. Two perpendicular side views are shown. Local resolutions are color coded for the TM region. **(D)** Representative EM map. Densities for TM1-TM6 of PfFNT are contoured at 5 σ. cryo-EM, cryogenic-electron microscopy; EM, electron microscopy; FSC, Fourier shell correlation; PfFNT, *P*. *falciparum* formate–nitrite transporter; TM, transmembrane.
(TIF)

S3 Fig Sequence alignment of PfFNT and homologs in pathogenic *Plasmodium* parasites and prokaryotic FNT family members. Secondary structural elements of PfFNT are shown above the sequence alignment. Invariant and highly conserved residues are shaded yellow and gray, respectively. The conserved residues for intracellular region interactions are indicated by purple circles under the sequences. The indicated sequences were aligned with ClustalW. PfFNT, *P*. *falciparum* formate–nitrite transporter.
(TIF)

S4 Fig Cryo-EM structural determination of PfFNT–MMV007839. **(A)** A representative cryo-EM micrograph of PfFNT- MMV007839. (B) A representative 2D classification average. **(C)** Flowchart for EM data processing of datasets. Details can be found in the Methods. cryo-EM, cryo-electron microscopy; EM, electron microscopy; PfFNT, *P*. *falciparum* formate–nitrite transporter.
(TIF)

S5 Fig Cryo-EM analysis of the PfFNT–MMV007839 structure. **(A)** Gold standard FSC curve for the 3D refinement of the overall structure of PfFNT–MMV007839. The raw data can be found in S3 Data. **(B)** Angular distribution of the particles for final reconstructions. **(C)** Local resolution of the PfFNT–MMV007839 complex. Local resolutions are color coded for the TM region. cryo-EM, cryogenic-electron microscopy; FSC, Fourier shell correlation; PfFNT, *P*. *falciparum* formate–nitrite transporter; TM, transmembrane.
(TIF)

S6 Fig Fitting of the hemiketal form of MMV007839 and inhibitor docking results. **(A)** Transformation between the hemiketal form and vinylogous acid form of MMV007839 in solvent. **(B) and (C)** Fitting of the hemiketal form and vinylogous acid form of MMV007839 to the ligand density. The density for MMV007839, shown as the pink mesh, is contoured at 7.5 σ.
(TIF)

S7 Fig Structural comparison of PfFNT proteins. **(A)** Comparison of the structures of PfFNT in apo state. The 5 protomers of PfFNT in apo state of the current study are distinguished by different colors. The reported PfFNT in apo state (PDB code: 6VQQ) is shown as cartoon and colored gray. **(B)** Comparison of the protomers of PfFNT in complex with MMV007839 complex. The PfFNT–MMV007839 complex in the current study and the reported PfFNT–MMV007839 complex (PDB code: 6VQR) are colored light pink and gray, respectively. The MMV007839 in the 2 structures are shown as stick and colored yellow and gray, respectively. Inhibitor binding residues are shown as sticks. Hydrogen bonds are shown as the blue dashed lines. PDB, Protein Data Bank; PfFNT, *P*. *falciparum* formate–nitrite transporter.
(TIF)

S1 Table Cryo-EM data collection and refinement statistics of PfFNT. cryo-EM, cryo-electron microscopy; PfFNT, *P*. *falciparum* formate–nitrite transporter.
(DOCX)

S2 Table ITC results summary. ITC, isothermal titration calorimetry.
(DOCX)

S1 Data The individual numerical values for the ITC results in Fig 2A, 2F, 2G, and 2H. ITC, isothermal titration calorimetry.
(XLSX)

S2 Data The individual numerical values for the channel passages calculated by HOLE in Fig 4B. (XLSX)

S3 Data The individual numerical values for the FSC curves in S2A and S5A Figs. FSC, Fourier shell correlation.
(XLSX)

## Abbreviations

AI: artificial intelligence
cryo-EM: cryogenic-electron microscopy
CTF: contrast transfer function
CTH: carboxyl-terminal helix
DDM: n-dodecyl-b-D-maltoside
ecFocA: *E*. *coli* FocA
EH: extracellular helix
FNT: formate–nitrite transporter
FocA: formate channel
FSC: Fourier shell correlation
GDN: glyco-diosgenin
hGLUT1: human glucose transporter 1
hMCT1: human monocarboxylate transporter 1
HSC: hydrosulphide ion channel
ITC: isothermal titration calorimetry
MMV: Medicine for Malaria Venture
NirC: nitrite channel
NTH: N-terminal helix
NTL: N-terminal loop
PfFNT: *P*. *falciparum* formate–nitrite transporter
PfHT1: *P*. *falciparum* hexose transporter 1
RMSD: root–mean–square deviation
SAR: structure–activity relation
stFocA: *S*. *typhimurium* FocA
TM: transmembrane
vcFocA: *V*. *cholera* FocA
