# Supplementary figures and images for: Structural characterization of the Plasmodium falciparum lactate transporter PfFNT alone and in complex with antimalarial compound MMV007839 reveals its inhibition mechanism
Source: PLoS Biol. 2021 Sep 9;19(9):e3001386. doi: 10.1371/journal.pbio.3001386 (PMC8428694; doi:10.1371/journal.pbio.3001386)

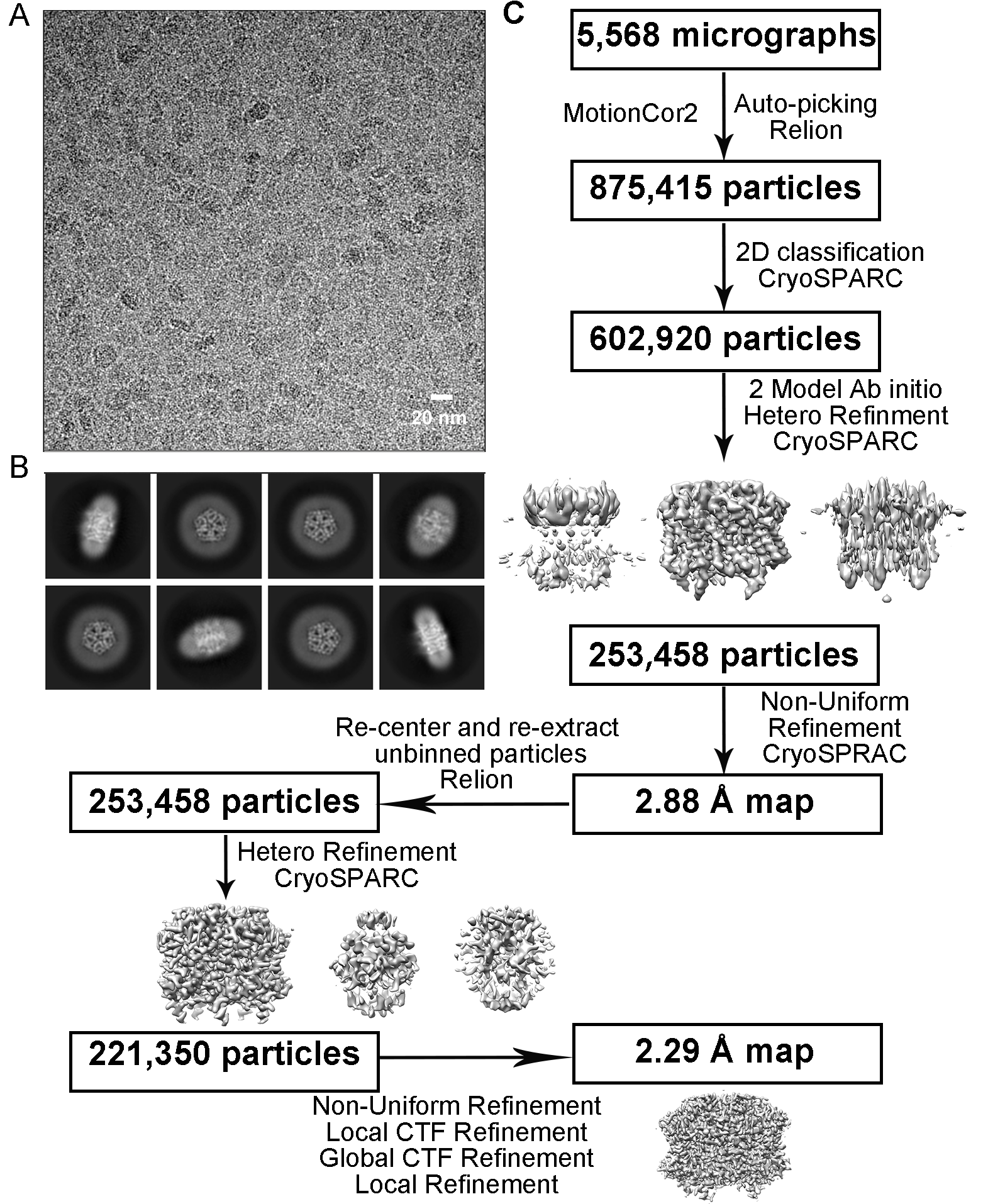

Supplement: S1 Fig — (A) A representative cryo-EM micrograph of PfFNT. (B) A representative 2D classification average. (C) Flowchart for EM data processing of PfFNT datasets. Details can be found in the Methods. cryo-EM, cryogenic-electron microscopy; EM, electron microscopy; PfFNT, P. falciparum formate–nitrite transporter. (TIF) [file pbio.3001386.s001.tif]

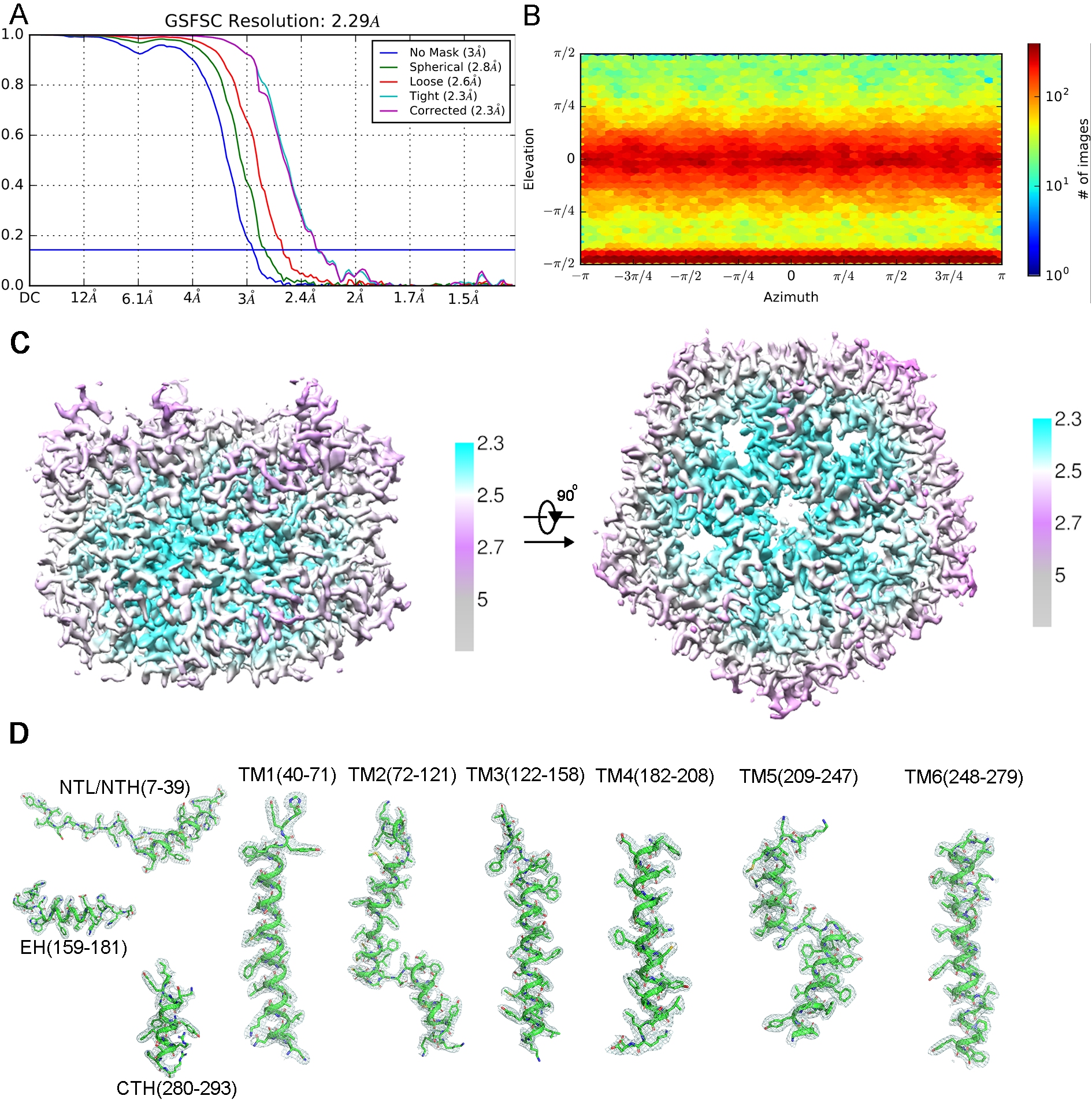

Supplement: S2 Fig — (A) Gold standard FSC curve for the 3D refinement of the overall structure of PfFNT. The raw data can be found in S3 Data. (B) Angular distribution of the particles used for the final reconstructions. (C) Local resolution of the PfFNT complex estimated by Chimera. Two perpendicular side views are shown. Local resolutions are color coded for the TM region. (D) Representative EM map. Densities for TM1-TM6 of PfFNT are contoured at 5 σ. cryo-EM, cryogenic-electron microscopy; EM, electron microscopy; FSC, Fourier shell correlation; PfFNT, P. falciparum formate–nitrite transporter; TM, transmembrane. (TIF) [file pbio.3001386.s002.tif]

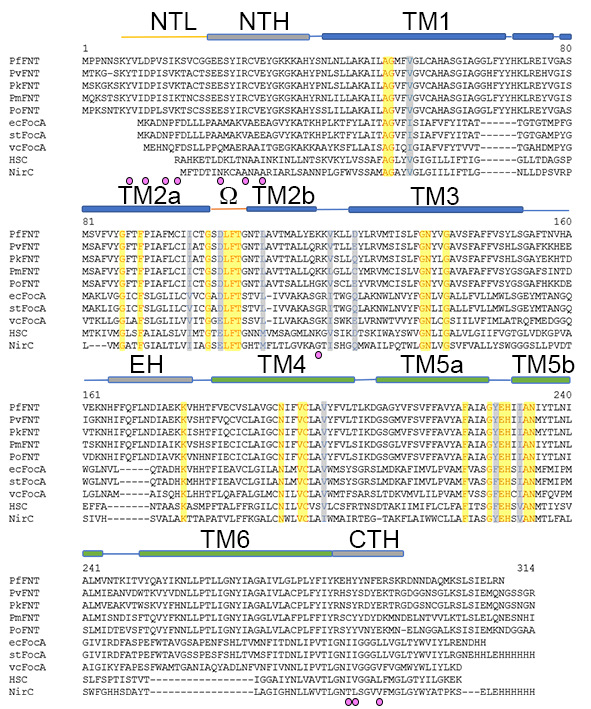

Supplement: S3 Fig — Secondary structural elements of PfFNT are shown above the sequence alignment. Invariant and highly conserved residues are shaded yellow and gray, respectively. The conserved residues for intracellular region interactions are indicated by purple circles under the sequences. The indicated sequences were aligned with ClustalW. PfFNT, P. falciparum formate–nitrite transporter. (TIF) [file pbio.3001386.s003.tif]

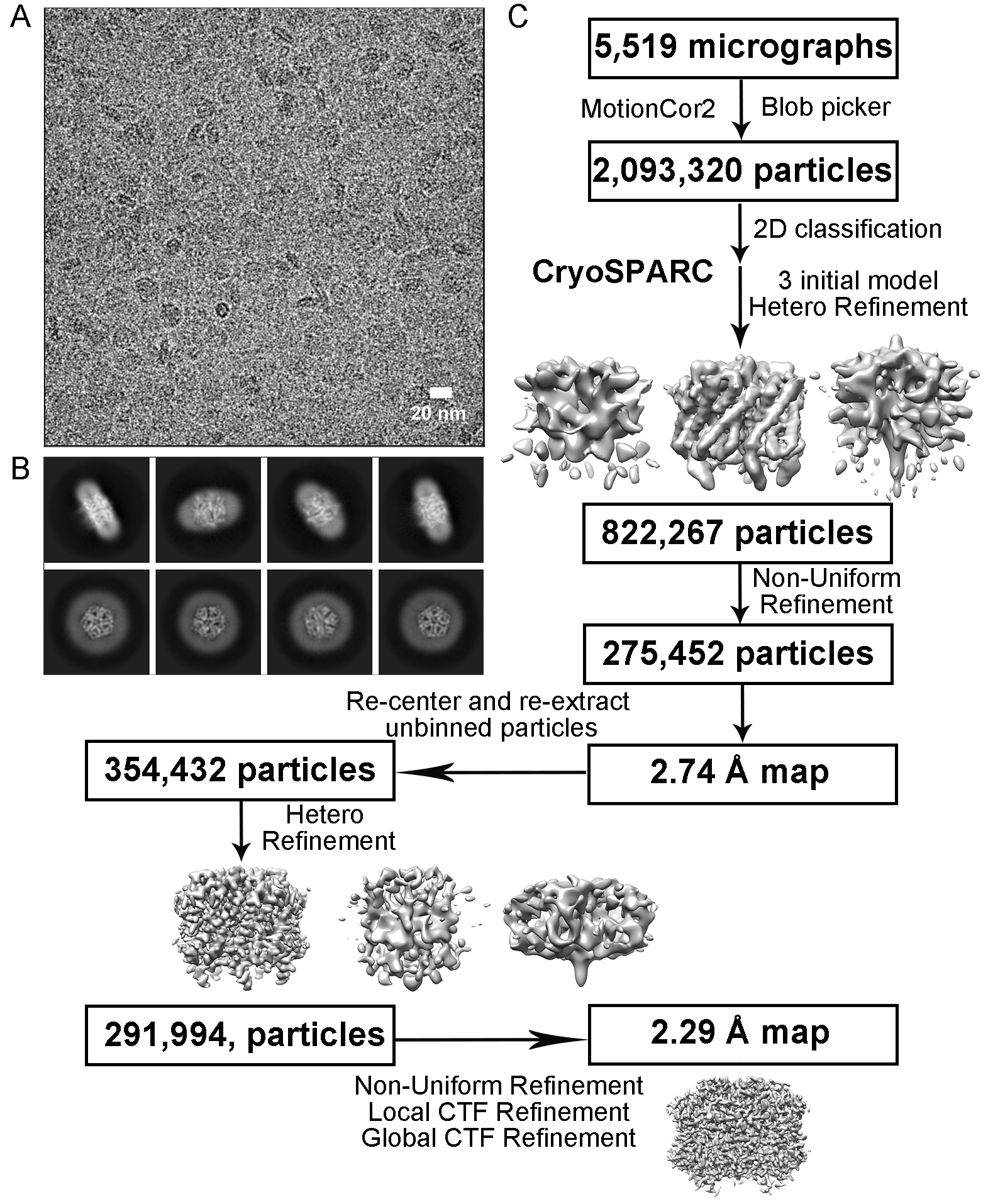

Supplement: S4 Fig — (A) A representative cryo-EM micrograph of PfFNT- MMV007839. (B) A representative 2D classification average. (C) Flowchart for EM data processing of datasets. Details can be found in the Methods. cryo-EM, cryo-electron microscopy; EM, electron microscopy; PfFNT, P. falciparum formate–nitrite transporter. (TIF) [file pbio.3001386.s004.tif]

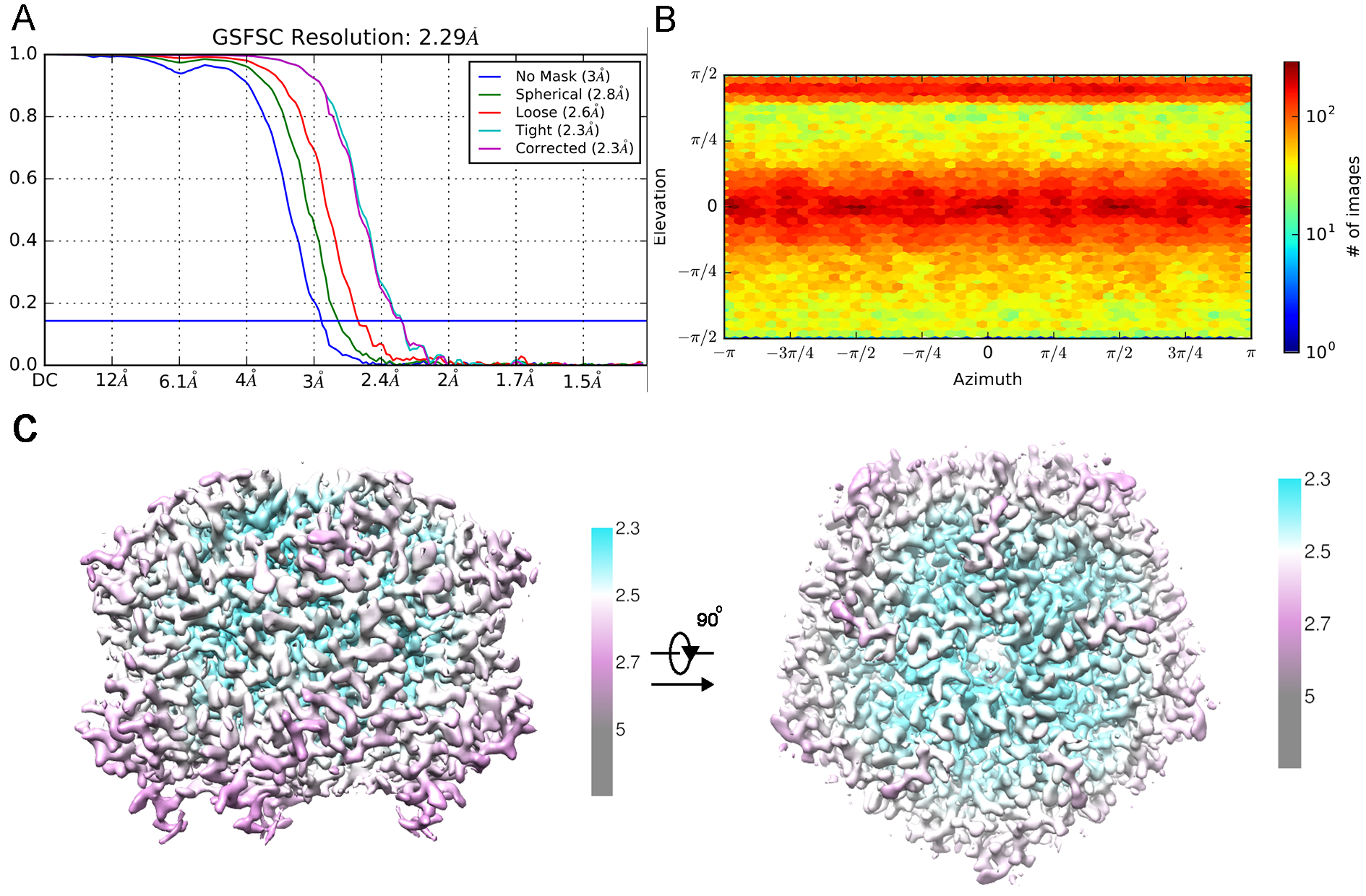

Supplement: S5 Fig — (A) Gold standard FSC curve for the 3D refinement of the overall structure of PfFNT–MMV007839. The raw data can be found in S3 Data. (B) Angular distribution of the particles for final reconstructions. (C) Local resolution of the PfFNT–MMV007839 complex. Local resolutions are color coded for the TM region. cryo-EM, cryogenic-electron microscopy; FSC, Fourier shell correlation; PfFNT, P. falciparum formate–nitrite transporter; TM, transmembrane. (TIF) [file pbio.3001386.s005.tif]

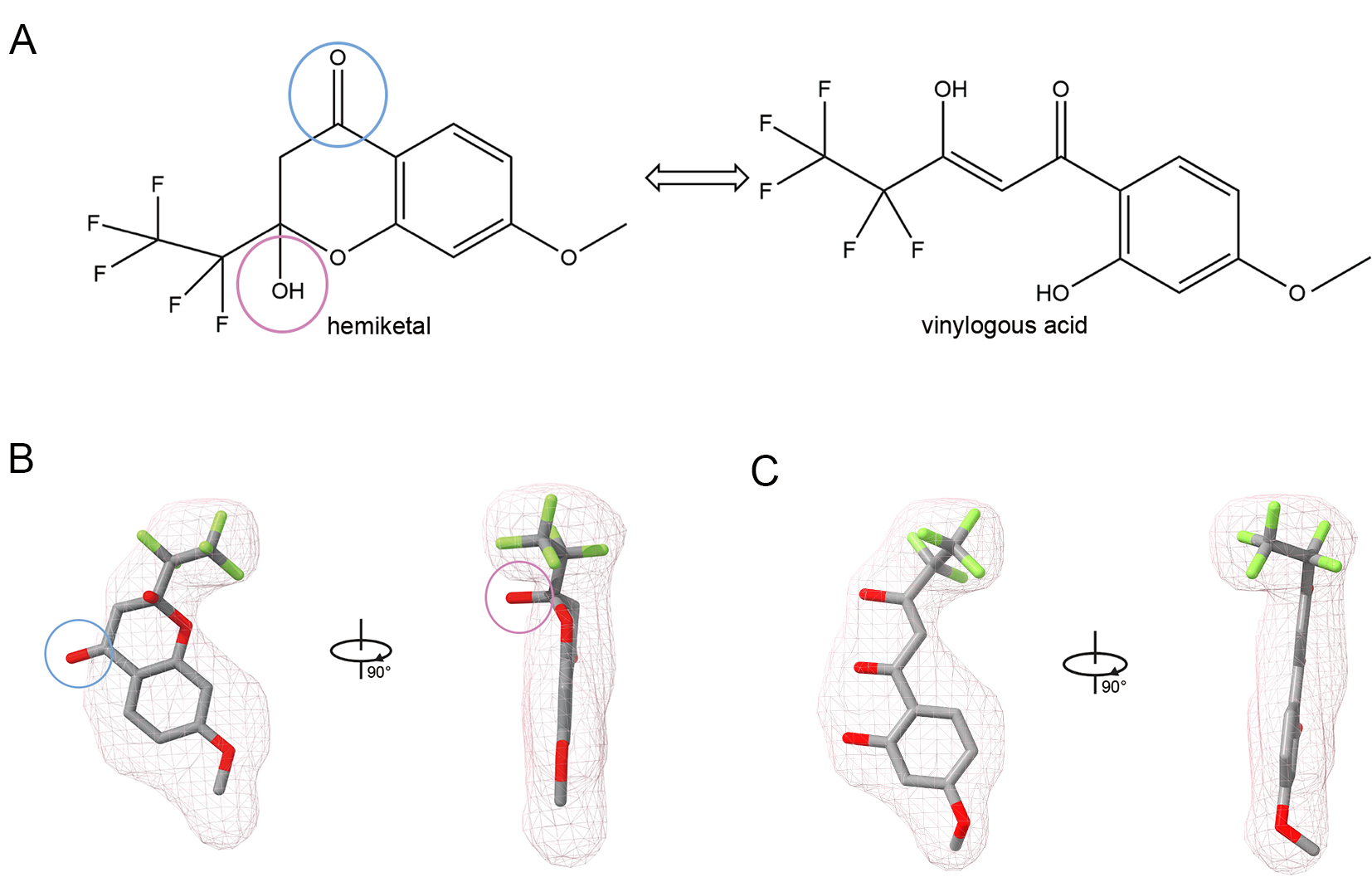

Supplement: S6 Fig — (A) Transformation between the hemiketal form and vinylogous acid form of MMV007839 in solvent. (B) and (C) Fitting of the hemiketal form and vinylogous acid form of MMV007839 to the ligand density. The density for MMV007839, shown as the pink mesh, is contoured at 7.5 σ. (TIF) [file pbio.3001386.s006.tif]

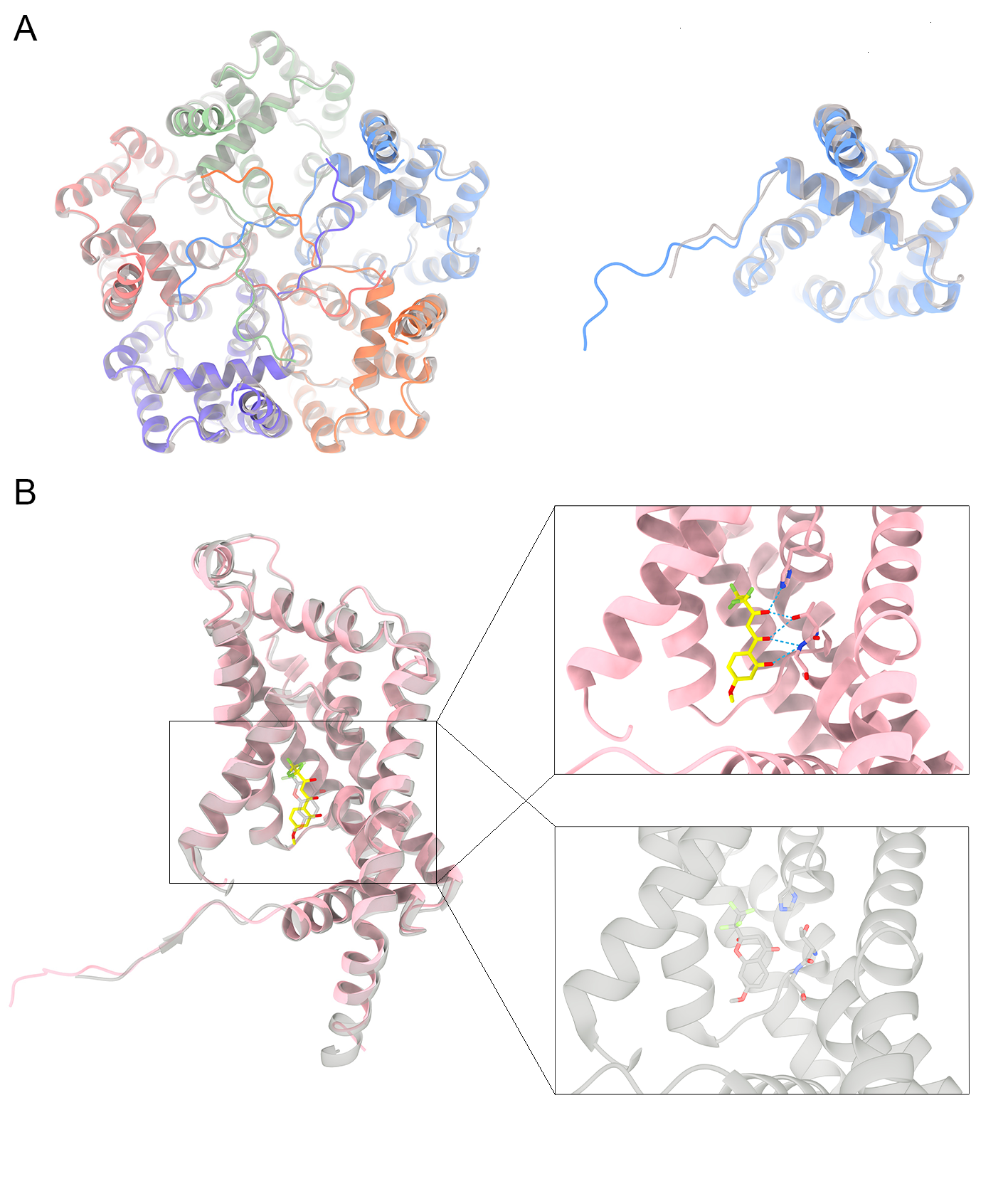

Supplement: S7 Fig — (A) Comparison of the structures of PfFNT in apo state. The 5 protomers of PfFNT in apo state of the current study are distinguished by different colors. The reported PfFNT in apo state (PDB code: 6VQQ) is shown as cartoon and colored gray. (B) Comparison of the protomers of PfFNT in complex with MMV007839 complex. The PfFNT–MMV007839 complex in the current study and the reported PfFNT–MMV007839 complex (PDB code: 6VQR) are colored light pink and gray, respectively. The MMV007839 in the 2 structures are shown as stick and colored yellow and gray, respectively. Inhibitor binding residues are shown as sticks. Hydrogen bonds are shown as the blue dashed lines. PDB, Protein Data Bank; PfFNT, P. falciparum formate–nitrite transporter. (TIF) [file pbio.3001386.s007.tif]
