# Supplementary material for: Structural characterization of the Plasmodium falciparum lactate transporter PfFNT alone and in complex with antimalarial compound MMV007839 reveals its inhibition mechanism
Source: PLoS Biol. 2021 Sep 9;19(9):e3001386. doi: 10.1371/journal.pbio.3001386 (PMC8428694; doi:10.1371/journal.pbio.3001386)
Supplement: S1 Table — cryo-EM, cryo-electron microscopy; PfFNT, P. falciparum formate–nitrite transporter. (DOCX) [file pbio.3001386.s008.docx]

**S1 Table. Cryo-EM data collection and refinement statistics of pfFNT.**

|  | **pfFNT-apo** | **pfFNT-MMV007839** |
| --- | --- | --- |
| **Data collection** |  |  |
| EM equipment | FEI Titan Krios | |
| Voltage (kV) | 300 | |
| Detector | K3 | |
| Pixel size (Å) | 0.6746 | 0.6746 |
| Electron dose (e-/Å^2^) | 50 | 50 |
| Defocus range (μm) | 1.5~2.0 | 1.5~2.0 |
| **Reconstruction** |  |  |
| Software | CRYOSPARC2.14 | |
| Number of used Particles | 221,350 | 291,994 |
| Symmetry | C5 | C5 |
| Map sharpening B-factor (Å^2^) | -82.4 | -89.0 |
| Final Resolution (Å) | 2.3 | 2.3 |
| **Model building and refinement** | | |
| Software | PHENIX & COOT | |
| Cell dimensions |  |  |
| a=b=c (Å) | 242.856 | 242.856 |
| α=β=γ (˚) | 90 | 90 |
| Protein residues |  |  |
| Total residue number | 1435 | 1435 |
| Residue number with side chain | 1435 | 1435 |
| Ligand  R.m.s deviations | —— | MMV007839 |
| Bonds length (Å) | 0.006 | 0.008 |
| Bonds Angle (˚) | 0.650 | 0.816 |
| Ramachandran plot statistics (％) |  |  |
| Preferred | 98.25 | 98.60 |
| Allowed | 1.75 | 1.4 |
| Outlier | 0.0 | 0.0 |
| Molprobity score | 1.23 | 1.82 |
