# Supplementary material for: Structural characterization of the Plasmodium falciparum lactate transporter PfFNT alone and in complex with antimalarial compound MMV007839 reveals its inhibition mechanism
Source: PLoS Biol. 2021 Sep 9;19(9):e3001386. doi: 10.1371/journal.pbio.3001386 (PMC8428694; doi:10.1371/journal.pbio.3001386)
Supplement: S2 Table — ITC, isothermal titration calorimetry. (DOCX) [file pbio.3001386.s009.docx]

**S2 Table ITC results summary**

| Protein | repeat1  K_d_ | | repeat2 | | repeat3 | K_d_  Mean ±SD (nM) | N  Mean ±SD |
| --- | --- | --- | --- | --- | --- | --- | --- |
| WT | 8.09 nM（N=0.95） | 4.98 nM（N=1.07） | | 8.47 nM（N=0.91） | | 7.18±1.91 | 0.98±0.08 |
| T106A | 68.7 nM（N=1.02） | 91.3 nM（N=1.04） | | 82.3 nM（N=1.13） | | 80.77±11.38 | 1.06±0.06 |
| H230A | UD | UD | | UD | | UD | UD |
| G107S | UD | UD | | UD | | UD | UD |
